# TLR-Mediated Inflammatory Responses to *Streptococcus pneumoniae* Are Highly Dependent on Surface Expression of Bacterial Lipoproteins

**DOI:** 10.4049/jimmunol.1401413

**Published:** 2014-08-29

**Authors:** Gillian Tomlinson, Suneeta Chimalapati, Tracey Pollard, Thabo Lapp, Jonathan Cohen, Emilie Camberlein, Sian Stafford, Jimstan Periselneris, Christine Aldridge, Waldemar Vollmer, Capucine Picard, Jean-Laurent Casanova, Mahdad Noursadeghi, Jeremy Brown

**Affiliations:** * Division of Infection and Immunity, University College London, London WC1E 6BT, United Kingdom;; † Centre for Inflammation and Tissue Repair, Division of Medicine, University College Medical School, Rayne Institute, London WC1E 6JF, United Kingdom;; ‡ Infectious Diseases and Microbiology Unit, University College London Institute of Child Health, London WC1N 1Eh, United Kingdom;; § Centre for Bacterial Cell Biology, Newcastle University Medical School, Newcastle upon Tyne NE2 4AX, United Kingdom;; ¶ Laboratory of Human Genetics of Infectious Diseases, Necker Branch, INSERM U980, Necker Medical School, University Paris Descartes, Sorbonne Paris Cité, Paris 75015, France;; ‖ Study Center for Primary Immunodeficiencies, Public Assistance-Paris Hospitals, Necker Enfants Malades Hospital, Paris 75743, France; and; # St. Giles Laboratory of Human Genetics of Infectious Diseases, Rockefeller Branch, The Rockefeller University, New York, NY 10065

## Abstract

*Streptococcus pneumoniae* infections induce inflammatory responses that contribute toward both disease pathogenesis and immunity, but the host–pathogen interactions that mediate these effects are poorly defined. We used the surface lipoprotein-deficient ∆*lgt* pneumococcal mutant strain to test the hypothesis that lipoproteins are key determinants of TLR-mediated immune responses to *S. pneumoniae*. We show using reporter assays that TLR2 signaling is dependent on pneumococcal lipoproteins, and that macrophage NF-κB activation and TNF-α release were reduced in response to the ∆*lgt* strain. Differences in TNF-α responses between *Δlgt* and wild-type bacteria were abrogated for macrophages from TLR2- but not TLR4-deficient mice. Transcriptional profiling of human macrophages revealed attenuated TLR2-associated responses to *∆lgt S. pneumoniae*, comprising many NF-κB–regulated proinflammatory cytokine and chemokine genes. Importantly, non-TLR2–associated responses were preserved. Experiments using leukocytes from IL-1R–associated kinase-4–deficient patients and a mouse pneumonia model confirmed that proinflammatory responses were lipoprotein dependent. Our data suggest that leukocyte responses to bacterial lipoproteins are required for TLR2- and IL-1R–associated kinase-4–mediated inflammatory responses to *S. pneumoniae*.

## Introduction

*Streptococcus pneumoniae* pneumonia, meningitis, and septicaemia are major causes of morbidity and mortality, responsible for >800,000 childhood deaths per year (1). *S. pneumoniae* infections are characteristically associated with inflammation (2), including a strong acute-phase response, rapid leukocyte recruitment to the site of infection, and compromise of endothelial or epithelial barriers. Although the inflammatory response is required for host immunity, excessive inflammation contributes toward serious complications of *S. pneumoniae* infection, such as neurologic damage, septic shock, and acute lung injury (3, 4). Characterizing the factors that drive the inflammatory response to *S. pneumoniae* will help explain the pathogenesis of pneumococcal infections and identify targets for novel therapies.

TLR are a key mechanism for innate immune sensing and are important for the host response to *S. pneumoniae*. Genetic deficiency of TLR signaling pathway proteins substantially increases the risk of invasive *S. pneumoniae* infections (5–7). Fifty-four percent of children with IL-1R–associated kinase 4 (IRAK-4) deficiency and 41% of patients with MyD88 deficiency have at least one episode of invasive pneumococcal disease (5, 8–10). Similarly, mice deficient in the TLR-signaling molecule MyD88 are highly susceptible to *S. pneumoniae* infections (11, 12). IRAK-4 deficiency largely prevents inflammatory responses to purified TLR ligands (6, 7), including expression of the cytokines TNF-α, IL-1β, and IL-6 that are important for host immunity to *S. pneumoniae* (13–15). Although innate immune recognition of *S. pneumoniae* is dependent on contributions from several TLRs, including TLR2, TLR4, and TLR9 (11, 16–24), the release of inflammatory cytokines such as TNF-α and IL-6 seems to be particularly dependent on TLR2 (19–21, 25). TLR2 contributes to the inflammatory response and control of infection in mouse models of meningitis and pneumonia (18, 22–24) and has additional effects that may affect disease development. First, the proinflammatory effects of TLR4 activation and of the *S. pneumoniae* toxin pneumolysin are partly dependent on synergistic activation of TLR2 (26, 27). Second, adaptive immune responses to *S. pneumoniae* can be impaired in TLR2-deficient mice (19, 28, 29). Third, TLR2-mediated respiratory epithelium responses to infection by *S. pneumoniae* increase tight junction breakdown and bacterial translocation across epithelial layers (30). Hence, identifying the *S. pneumoniae* TLR2 ligands is necessary for understanding of the molecular mechanisms contributing to disease pathogenesis by this pathogen.

Known TLR2 ligands include peptidoglycan (PGN) and lipoteichoic acid (LTA), both components of the Gram-positive cell wall (31, 32). The *S. pneumoniae* cell wall is highly proinflammatory, suggesting PGN and LTA could be major TLR2 agonists (33–35). However, cell wall–dependent inflammation is also mediated by NOD recognition of PGN (36, 37), and purified *S. pneumoniae* LTA does not stimulate IL-8 production by HEK293 cells transfected with TLR2 (38). Hence, cell wall products may not be dominant TLR2 agonists for *S. pneumoniae*. Bacterial lipoproteins are important TLR2 ligands for other Gram-positive pathogens (32, 39, 40), and *S. pneumoniae* expresses a large number of lipoproteins, many of which are important for bacterial virulence (41–46). However, the importance of *S. pneumoniae* lipoproteins for TLR2-mediated immune recognition and proinflammatory responses has not been investigated. We have used a *S. pneumoniae* mutant strain with markedly reduced surface lipoprotein content due to deletion of the lipoprotein diacylglyceryl transferase gene *lgt* (45) to assess the contribution of lipoproteins to TLR-dependent inflammatory responses to *S. pneumoniae*. The effects of lipoproteins on macrophage responses to *S. pneumoniae* were characterized in detail using transcriptome analysis and by measuring important proinflammatory cytokine responses of mouse macrophages with deletions affecting the TLR pathway and leukocytes from patients with IRAK-4 deficiency.

## Materials and Methods

### Ethics statement

Experiments using human cells were approved by the joint University College London/University College Hospitals National Health Service Trust Human Research Ethics Committee, and written informed consent was obtained from all participants. All animal experiments were approved by the University College London Biological Services Ethical Committee and the United Kingdom Home Office (Project License PPL70/6510) and performed according to United Kingdom national guidelines for animal use and care.

### Bacterial strains and growth conditions

The *S. pneumoniae* strain, TIGR4, was a gift of J. Weiser (University of Pennsylvania, Philadelphia, PA). The *∆lgt* strains were obtained by in-frame deletion of *lgt* (Sp1412) from wild-type (WT) TIGR4, D39, and *∆pab* TIGR4 strains, as previously described (45, 47). Mutant strains were genome sequenced by the Wellcome Trust Centre for Human Genetics (Oxford, U.K.) using an Illumina MiSeq sequencer. Sequences were assembled using Velvet, annotated using Prokka, and mapped to the published TIGR4 and D39 (R00000036) reference genomes. Bases and single-nucleotide variants were identified using the SAMtools “mpileup” command and BCFtools. Sites were filtered to a minimum depth of five reads at each and single-nucleotide variant quality of 25, and the Integrated Genome Viewer was used to visualize mapping and coverage. *S. pneumoniae* was cultured overnight at 37°C in 5% CO_2_ on Columbia agar (Oxoid) supplemented with 5% horse blood (TCS Biosciences). Working stocks grown to an OD of 0.4 (∼10^8^ CFU/ml) were made using Todd-Hewitt broth supplemented with 0.5% yeast extract and stored at −80°C in 10% glycerol as single-use aliquots. CFU were confirmed by colony counting of log_10_ serial dilutions of bacteria cultured overnight on 5% Columbia blood agar. Chloramphenicol (10 μg ml^−1^) and kanamycin (500 μg ml^−1^) were added to blood agar plates where appropriate.

### Preparation of bacterial lysates and Triton X-114 extraction of lipoproteins

Bacterial lysates were made using mid log-phase growth *S. pneumoniae* cells by addition of 0.1% deoxycholate (Sigma-Aldrich) in PBS for 30 min at 37°C and sonication with a Soniprep 150 (Sanyo) ultrasonicator. Membrane-associated proteins were extracted from lysates by Triton X-114 extraction, as described previously (48, 49), washed, and diluted 1:2 in PBS prior to solubilization in Laemmli sample buffer for SDS-PAGE and visualization using Coomassie brilliant blue (Sigma-Aldrich) staining.

### Cell isolation and culture

Blood samples were obtained from healthy volunteers or IRAK-4–deficient subjects homozygous for the Q293X mutation for isolation of PBMC or production of monocyte-derived macrophage (MDM) by differentiation with M-CSF, as previously described (50). Bone marrow was extracted from 6- to 8-wk-old C57BL/6 WT, *TLR2*^−/−^, *TLR4*^−/−^ (Jackson ImmunoResearch Laboratories), or *Myd88/trif*^−/−^ mice (gift of S. Akira, Department of Host Defense, Research Institute for Microbial Diseases, Osaka University, Suita, Osaka, Japan) and differentiated into bone marrow–derived macrophages (BMDM) for 7 d in L929-conditioned medium using standard protocols (51, 52). The RAW 264.7 murine macrophage cell line was cultured as adherent cells in RPMI 1640 (Life Technologies) supplemented with 2 mM l-glutamine (Invitrogen) and 10% FBS (Life Technologies).

### PGN purification and structural analysis

Pneumococcal cell wall (PGN–teichoic acid complex) and PGN were prepared, as described (53), and muropeptide was released by digesting PGN with cellosyl (provided by Hoechst). Muropeptides were reduced with sodium borodydride and analyzed by high-pressure liquid chromatography, as described (53). The peaks were assigned by comparing their retention time with the retention time of known muropeptides obtained from strain R6, whose muropeptide profile is similar to that of strain TIGR4. Peak 1 (Fig. 3) was collected and analyzed by electrospray mass spectrometry, as described (53, 54).

### Innate immune stimulation, cytokine measurements, and NF-κB nuclear translocation

HEK TLR2 reporter assays were stimulated with *S. pneumoniae* for 16 h, according to the manufacturer’s instructions (Invivogen). White cells were stimulated as follows for collection of supernatants, RNA isolation, or NF-κB translocation assays: MDM and PBMC stimulated for 1–24 h with *S. pneumoniae* strains at a multiplicity of infection (MOI) of 5–50 or Pam_2_CSK_4_ (100 ng/ml; Axis-Shield); RAW 264.7 cells stimulated with different *S. pneumoniae* strains at a MOI of 5 or 10 μl (in a total volume of 300 μl) Triton X-114 extracts for up to 4 h (47); BMDM stimulated with *S. pneumoniae* bacterial strains in DMEM without any supplements for 4 h at a MOI of 5. Cytokine levels were measured in cell culture supernatants using ELISA (R&D Systems or eBioscience), or the Luminex or MSD platforms, according to the manufacturer’s instructions. NF-κB activation was assessed by quantifying nuclear RelA using the MSD system, according to the manufacturer’s instructions, or using immunofluorescence of MDMs to obtain nuclear:cytoplasmic ratios of NF-κB Rel A (p65) staining as a marker of NF-κB nuclear translocation (50, 55).

### Transcriptional profiling by cDNA microarray and quantitative PCR

Total RNA was purified from cell lysates using the RNEasy mini kit (Qiagen). Samples were processed for Agilent microarrays, and data were normalized, as previously described (56). Microarray data are available in the ArrayExpress database (www.ebi.ac.uk/arrayexpress) under accession number E-MTAB-1541. For quantitative PCR (qPCR), cDNA was synthesized using the qScript cDNA Supermix kit (Quanta BioSciences), and qPCR of selected genes was performed using the following inventoried TaqMan assays (Applied Biosystems): *IL23* (Hs00372324_m1), *IL1β* (Hs01555410_m1), *IL6* (Hs00985639_m1), and *TNFα* (Hs00174128_m1). *PTSG2* expression was quantified using the following: forward primer, 5′-CGGTCCTGGCGCTCAG-3′; reverse primer, 5′-CCGGGTACAATCGCACTTATACTG-3′; and probe, 5′-CCATACAGCAAATCCTT-3′ (Applied Biosystems). Expression levels of target genes were normalized to *GAPDH*, as previously described (57).

### Animal model of lung infection

Six- to 8-wk-old outbred CD1 female white mice (Charles Rivers Breeders) were inoculated intranasally with 1 × 10^7^ CFU in an inoculum volume of 50 μl under halothane (Zeneca) general anesthesia. Bronchoalveolar lavage fluid (BALF) samples were obtained from culled mice 4 h postinfection for cytokine assays using ELISAs (R&D Systems), bacterial CFU by colony counting of serial dilutions plated onto Columbia blood agar plates containing 5 μg ml^−1^ gentamicin, and total cell counts using a manual hemocytometer (Gova Glasstic slides; Hycor Biomedical UK) under light microscopy (original magnification ×20) following 1:2 dilution of BALF in 0.001% crystal violet in acetic acid.

### Statistical analysis

Data that would be normally distributed (results of macrophage cytokine analyses and qPCR) were analyzed using paired (for data obtained from matched samples) or unpaired (for data obtained from unmatched samples) *t* tests. Nonnormally distributed data (data from mouse experiments and NF-κB nuclear translocation data) were compared using the Mann–Whitney *U* test. Principal component (PC) analysis was used to compare global gene expression profiles, as previously described, and *t* tests were used to identify significant gene expression differences (*p* < 0.05) between samples using MultiExperiment Viewer v4.6.0 (57). Transcriptional regulation of specific gene signatures was assessed by analysis of single transcription factor binding site enrichment analysis (http://opossum.cisreg.ca/oPOSSUM3/). Pathway and Gene Ontology overrepresentation analysis was performed using InnateDB (http://www.innatedb.com/).

## Results

### TLR2 signaling in response to *S. pneumoniae* is dependent on lipoproteins

**FIGURE 1.**
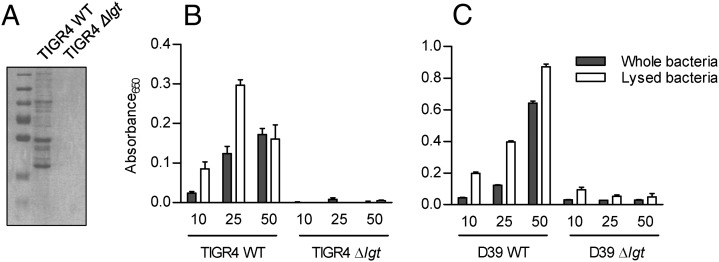
TLR2 activation by *S. pneumoniae* is dependent on surface lipoproteins. (**A**) Coomassie blue staining of Triton-X–extracted membrane proteins from the WT (TIGR4) and TIGR4*Δlgt* strains separated by SDS-PAGE, confirming markedly reduced lipoprotein content for the *Δlgt* strain. A molecular mass marker (15–80 kDa) is also shown. (**B** and **C**) Mean relative absorbance (OD_650_) of supernatants from a TLR2/HEK reporter cell line incubated for 16 h with different MOI of live (empty columns) or lysed (gray columns, using deoxycholate) (B) TIGR4*Δlgt* or TIGR4 and (C) D39*Δlgt* or D39 *S. pneumoniae*. Error bars represent SEMs, and *n* = 3 with data representative of repeated experiments.

Triton-X extracts of the TIGR4 Δ*lgt S. pneumoniae* strain confirmed that this strain has almost no detectable lipoproteins, similar to the previously described 0100993 Δ*lgt* strain (Fig. 1A) (45). A reporter assay was used to assess whether loss of lipoproteins significantly affected TLR2 signaling by *S. pneumoniae* (Fig. 1B). Live whole TIGR4 WT bacteria stimulated a strong TLR2-dependent signal, whereas live Δ*lgt* bacteria did not cause any significant response even with high multiplicities of infection (MOI). Lysed bacteria gave similar results, demonstrating that impaired growth or failure of release of nonlipoprotein TLR2 ligands such as PG and LTA was not responsible for the lack of TLR2 response to the Δ*lgt* bacteria. We have not been able to complement the Δ*lgt* mutation (45); hence, to confirm linkage of the loss of TLR2 responses to the mutation, we transferred the mutation to the D39 *S. pneumoniae* strain and obtained similar results to the TIGR4 strain, with almost complete loss of TLR2-dependent signaling to both live and lysed D39Δ*lgt* (Fig. 1C). Whole-genome sequencing of the Δ*lgt* TIGR4 and D39 *S. pneumoniae* strains was performed to identify unexpected mutations that might confound the results (Supplemental Table I). Both the D39 and TIGR4 Δ*lgt* strains contained the expected complete deletion of *lgt* (replaced by the *kan* antibiotic resistance gene), as well as two synonymous single nucleotide polymorphisms (SNPs) in the gene immediately downstream of *lgt* (Spd_1244 and Sp_1413, respectively) that encodes a Hpr(Ser) kinase/phosphatase. In addition, the Δ*lgt* TIGR4 strain contained one SNP in a noncoding intergenic region, and one nonsynonymous SNP changing a histidine for an arginine at position 336 of a 413-aa residue protein encoded by Sp_2175 (*dltB*), predicted to be an alanine exporter involved in lipoteichoic acid synthesis. These data indicate that loss of TLR2 stimulation was linked to deletion of *lgt* and suggest that lipoproteins are major *S. pneumoniae* TLR2 ligands.

### Mouse macrophage TNF-α response to *S. pneumoniae* is dependent on lipoproteins

**FIGURE 2.**
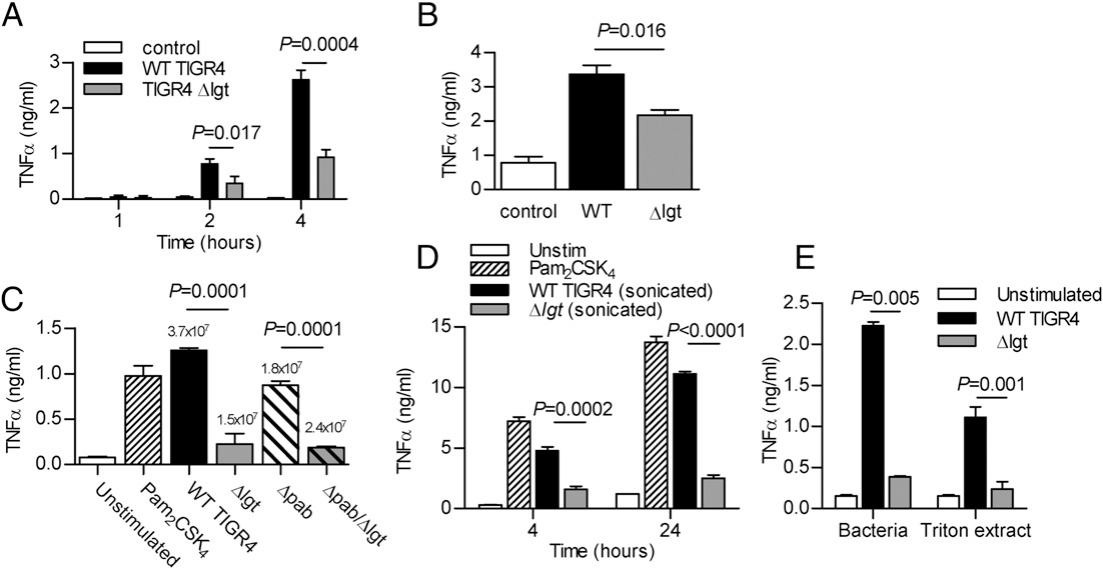
Macrophage TNF-α responses are dependent on *S. pneumoniae* lipoproteins. (**A** and **B**) Time course of TNF-α concentrations (measured by ELISA) in RAW cell culture supernatants after incubation with TIGR4 or TIGR4*Δlgt S. pneumoniae* (MOI 5). (**C**) TNF-α concentrations in RAW cell culture supernatants after 4-h incubation with Pam_2_CSK_4_, WT, TIGR4*Δlgt*, TIGR4*ΔpabB*, or TIGR4*ΔlgtΔpabB S. pneumoniae*. Bacterial CFU after 4-h incubation are stated above each column. (**D**) TNF-α concentrations in RAW cell culture supernatants after incubation for 4 and 24 h with the TLR agonist Pam_2_CSK_4_, or sonicated TIGR4 or TIGR4*Δlgt S. pneumoniae*. (**E**) TNF-α concentrations in RAW cell culture supernatants after 4-h incubation with TIGR4 or TIGR4*Δlgt S. pneumoniae* or their corresponding 3.3% Triton X-114 lipoprotein. For all panels, *n* = 3–4 and is representative of repeated experiments, data are presented as means, error bars represent SEMs, and *p* values were obtained using unpaired *t* tests.

To assess the significance of lipoprotein-dependent TLR2 signaling on the strength of the inflammatory response to *S. pneumoniae*, TNF-α secretion by the RAW mouse macrophage cell line was compared after incubation with WT or Δ*lgt* TIGR4. Significant production of TNF-α was evident after 2-h incubation with WT *S. pneumoniae* (Fig. 2A), but was significantly attenuated in response to the Δ*lgt* strain at 2, 4, and 24 h (Fig. 2A, 2B). Loss of lipoproteins affects *S. pneumoniae* growth (45), and after 4-h incubation with RAW cells there are ∼60% fewer Δ*lgt* bacteria/ml than the WT strain (Fig. 2C). However, the reduced TNF-α response to the Δ*lgt* strain persisted when RAW cells were stimulated with sonicated bacteria (Fig. 2D), and after transfer of the *lgt* mutation to the Δ*pabB* strain (Fig. 2C), an auxotrophic mutant that replicates poorly without addition of exogenous para-aminobenzoic acid (47). Furthermore, there was a similar scale reduction in the RAW cell TNF-α response to the Δ*lgt* strain when RAW cells were stimulated with Triton-X extracts for 4 h, providing further evidence of a direct effect of lipoproteins on inflammatory responses (Fig. 2E).

### The Δlgt mutation has no effect on PGN structure

**FIGURE 3.**
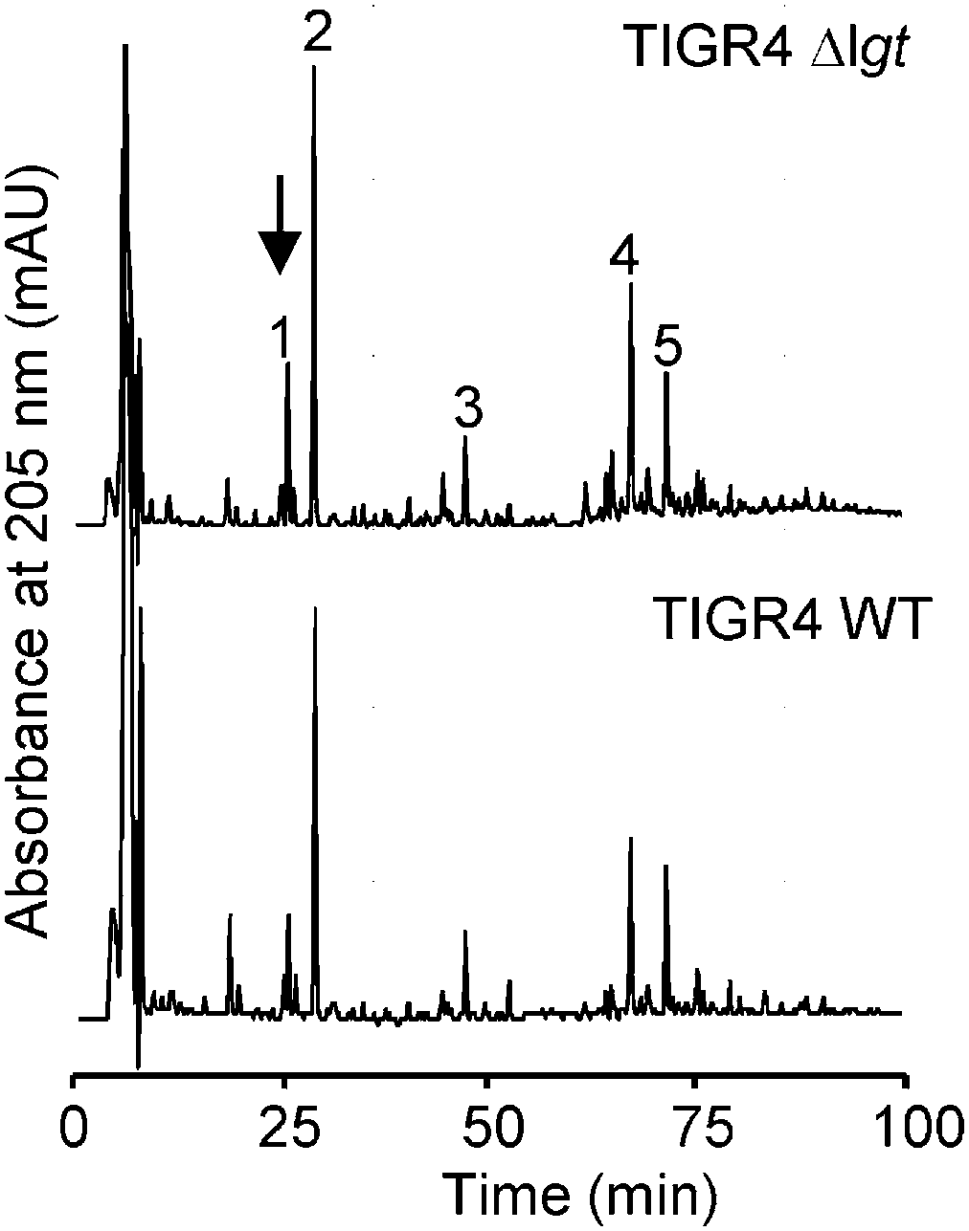
Normal muropeptide profile for PGN from the TIGR4Δ*lgt* strain. HPLC analysis of muropeptides isolated from cell wall preparations of the TIGR4 and TIGR4Δ*lgt* strains. Major muropeptides are indicated by numbers, as follows: 1, Glc*N*-Mur*N*Ac-L-Ala-D-iGln-L-Lys; 2, Glc*N*Ac-Mur*N*Ac-L-Ala-D-iGln-L-Lys; 3, Glc*N*Ac-Mur*N*Ac-L-Ala-D-iGln-L-Lys-L-Ser-L-Ala; 4, Glc*N*Ac-Mur*N*Ac-L-Ala-D-iGln-L-Lys-D-Ala-L-Lys-D-iGln-L-Ala-Mur*N*Ac-Glc*N*Ac; 5, Glc*N*Ac-Mur*N*Ac-L-Ala-D-iGln-L-Lys-L-Ser-L-Ala-D-Ala-L-Lys-D-iGln-L-Ala-Mur*N*Ac-Glc*N*Ac. The structure of peak 1 (arrow) was confirmed by mass spectrometry. Glc*N*, glucosamine; Glc*N*Ac, *N*-acetylglucosamine; Mur*N*Ac, *N*-acetylmuramic acid.

As the PGN synthesis enzyme PGN deacetylase is a hypothetical lipoprotein, PGN structure could potentially be affected by deletion of *lgt* (58). However, the muropeptide profile of the Δ*lgt* TIGR4 strain did not significantly differ from that of WT TIGR4, and both were similar to the reported muropeptide profile of strain R6 (an unencapsulated derivative of D39) (53). Importantly, PGN from Δ*lgt* TIGR4 contained peaks corresponding to muropeptide with deacetylated Glc*N*Ac residues, which are generated by PGN deacetylase (53, 58). To further confirm the presence of deacetylated muropeptides in the Δ*lgt* TIGR4 sample, the main deacetylated monomer (peak 1, Fig. 3) was analyzed by mass spectrometry (MS). The obtained neutral mass of 783.3880 Da is consistent with the theoretical mass of 783.3862 Da for the deacetylated disaccharide tripeptide (Glc*N*-Mur*N*Ac-L-Ala-D-iGln-L-Lys). The MS/MS fragmentation pattern of this signal showed the expected loss of a dehydrated glucosamine residue (160.9152 Da, theoretical 161.0688). Therefore, there are no significant alterations in PGN structure that could account for the effects of the Δ*lgt* mutation on inflammatory responses to *S. pneumoniae*.

### *S. pneumoniae* lipoprotein effects on macrophage TNF-α responses are TLR2 dependent

**FIGURE 4.**
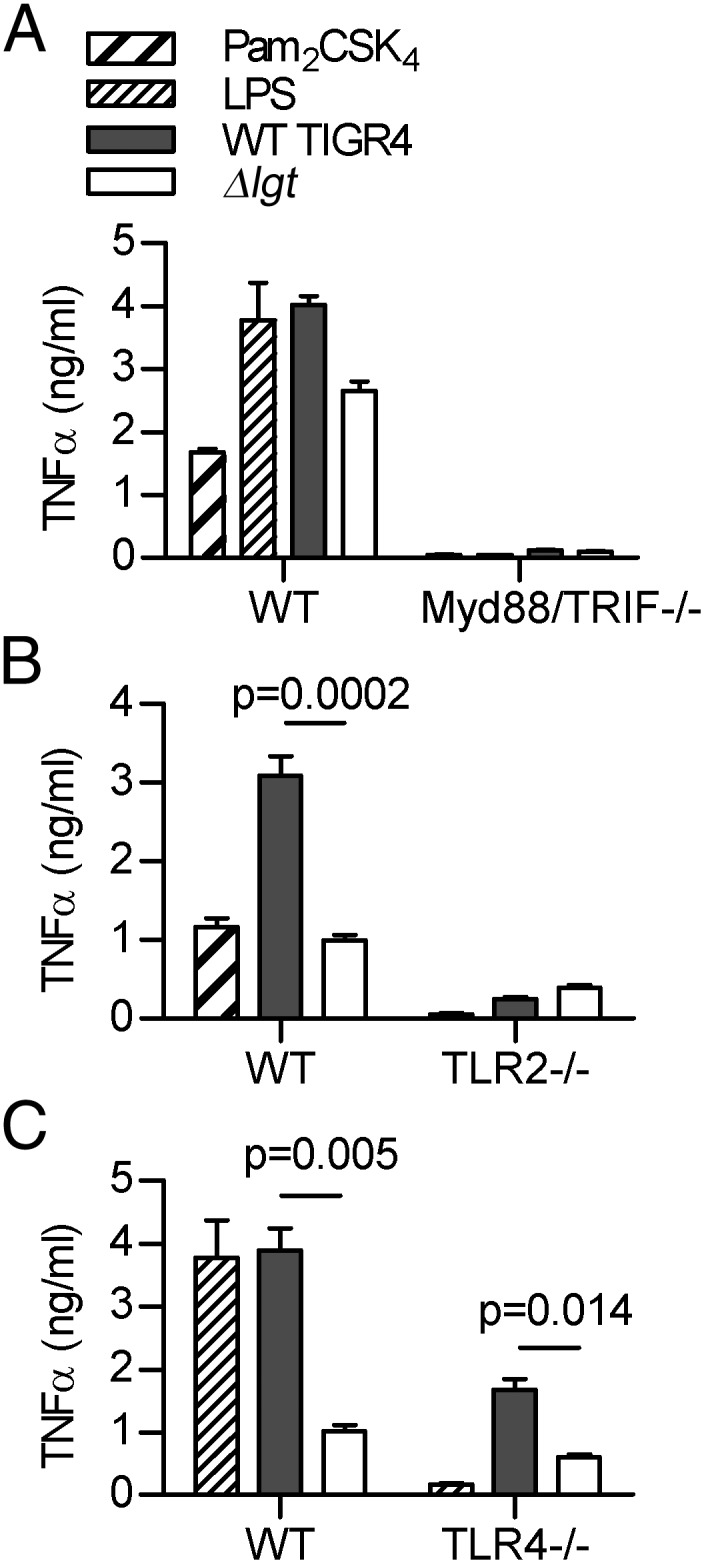
*S. pneumoniae* lipoproteins induce proinflammatory cytokines via TLR2 pathway activation. Mean TNF-α concentrations in cell culture supernatants from BMDMs obtained from C57BL/6 mice incubated for 4 h with the TLR agonists (LPS and/or Pam_2_CSK_4_), or TIGR4 or TIGR4*Δlgt S. pneumoniae*. (**A**) Results for BMDMs obtained from WT or *Myd88/trif^−^*^/−^ mice. (**B**) Results for BMDMs obtained from WT or *TLR2^−^*^/−^ mice. (**C**) Results for BMDMs obtained from WT or *TLR4^−^*^/−^ mice. For all panels, *n* = 3–4 and is representative of repeated experiments, error bars represent SEMs, and *p* values were obtained using unpaired *t* tests.

To investigate whether the differences in TNF-α responses between the WT and *Δlgt* strains were attributable to specific TLRs, TNF-α responses by BMDMs from WT, *tlr2^−/−^*, *tlr4^−/−^*, or *Myd88/trif^−/−^* mice were also evaluated. There was no significant TNF-α release by BMDMs from *Myd88/trif^−/−^* mice incubated with either the WT or *Δlgt* strain, showing the necessity of TLR signaling for the TNF-α response to *S. pneumoniae* (Fig. 4A). WT TIGR4 induced significantly greater TNF-α secretion by BMDMs from WT mice than the Δ*lgt* strain. TNF-α release by BMDMs from *tlr2^−/−^* mice was strongly reduced in comparison with BMDMs from WT mice, although there was still a residual response compared with *Myd88/trif^−/−^* BMDMs (Fig. 4A, 4B). Importantly, for BMDMs from *tlr2^−/−^* mice, there was no increased TNF-α response to WT TIGR4 compared with Δ*lgt* bacteria, indicating that the increase seen with BMDMs from WT mice was TLR2 dependent. In contrast, although TNF-α production by BMDMs from *tlr4^−/−^* mice was reduced in response to both strains compared with BMDMs from WT mice, WT TIGR4 still induced significantly higher levels of TNF-α secretion than the Δ*lgt* strain (Fig. 4C). These results demonstrate that the TLR2 but not the TLR4 response was dependent on bacterial lipoproteins.

### Loss of lipoproteins attenuates NF-κB activation in response to *S. pneumoniae*

**FIGURE 5.**
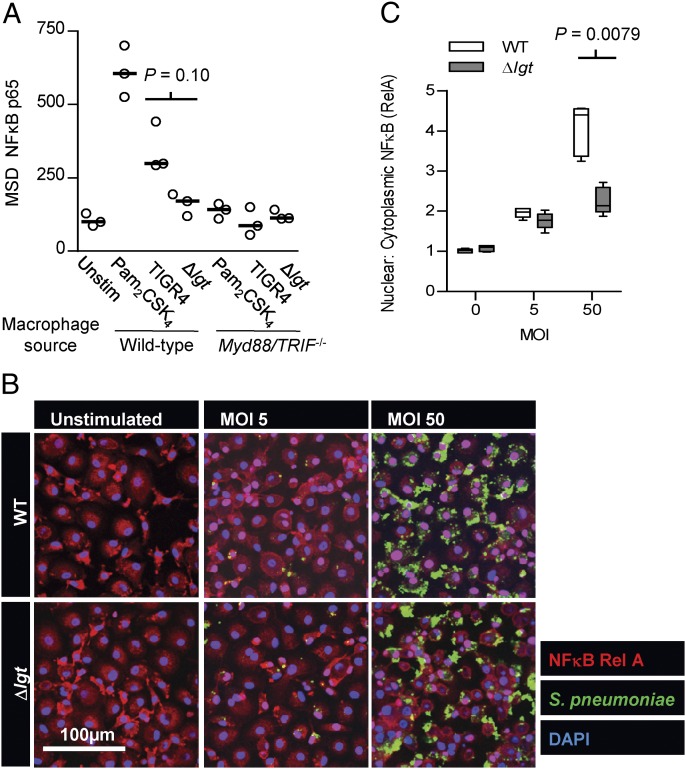
Pneumococcal lipoproteins are important determinants of TLR-induced NF-κB activation. (**A**) Quantification of nuclear NF-κB p65 using MSD (arbitrary units) for BMDMs from C57BL/6 WT and *Myd88/trif^−^*^/−^ mice incubated for 1 h with *Δlgt* and TIGR4 *S. pneumoniae* (MOI of 5). Each symbol represents results for one well, the bar represents median values, and the *p* value for comparison of TIGR4 and *Δlgt* strains incubated with WT BMDMs was obtained using a Mann–Whitney *U* test. (**B**) Confocal immunofluorescence images of NF-κB RelA in human MDM from five different donors stimulated for 2 h with WT (TIGR4) or lipoprotein-deficient (*Δlgt*) *S. pneumoniae* (MOI 5 or 50) showing diminished nuclear translocation of RelA (pink nuclei) for cells incubated with the *Δlgt* strain. (**C**) Quantitative image analysis of the median (IQR) ratio of nuclear to cytoplasmic RelA staining (*p = 0.0079, Mann–Whitney *U* test).

We then assessed whether loss of TLR2 signaling in response to the Δ*lgt* strain affected activation of the key proinflammatory transcriptional regulator NF-κB (59, 60) in macrophages. Quantifying NF-κB Rel A in nuclear extracts showed there was a significant increase in BMDM NF-κB activation by WT TIGR4 that was largely abrogated in macrophages from *Myd88/trif^−/−^* mice or in WT BMDMs infected with the Δ*lgt* strain (Fig. 5A). Similarly, NF-κB activation was significantly attenuated in response to the Δ*lgt* strain compared with WT TIGR4 when assessed using a confocal assay to quantify nuclear translocation of NF-κB Rel A in human MDMs (Fig. 5B, 5C). These data suggest both mouse and human macrophage NF-κB activation in response to *S. pneumoniae* is dependent on TLR recognition of lipoproteins.

### Comparison of the macrophage transcriptome to the TIGR4 and Δlgt strains

**FIGURE 6.**
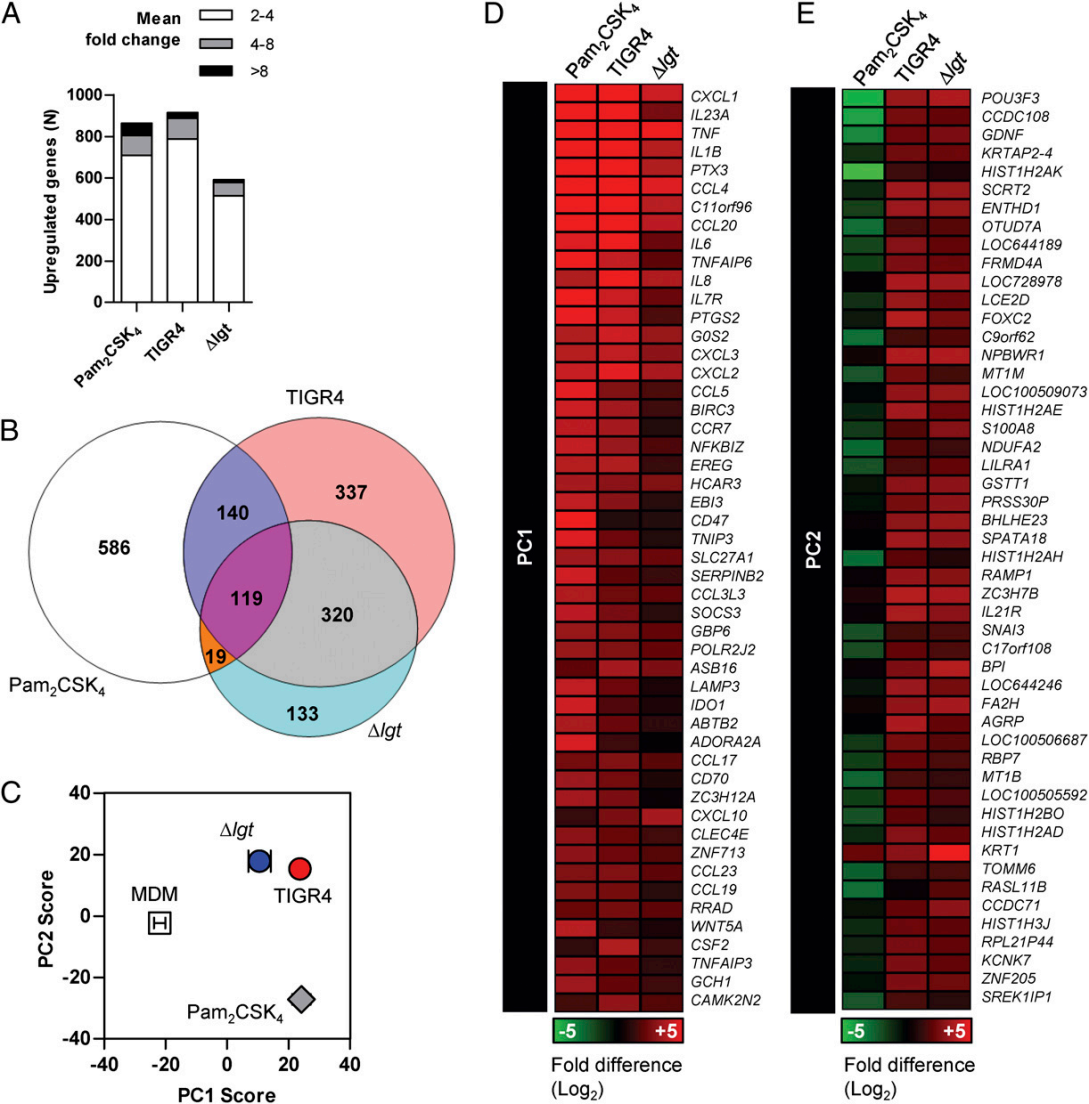
Attenuated TLR2-associated macrophage transcriptional responses in response to lipoprotein-deficient *S. pneumoniae*. Comparison of MDM genome-wide transcriptional responses from at least three donors after 4-h stimulation with Pam_2_CSK_4_, WT (TIGR4), or *Δlgt S. pneumoniae*. (**A**) Total number of genes upregulated at least >2-fold. (**B**) Venn diagram of the overlap between the genes upregulated >2-fold by each stimulus. (**C**) PC analysis showing quantitative differences in expression levels of cocorrelated gene signatures in the global gene expression profile. The graph plots PC1 and PC2, responsible for the greatest differences, for each condition (data points represent mean ± SEM of ≥3 separate experiments). (**D** and **E**) The gene expression heat map shows the transcriptional response (compared with unstimulated MDM) for the top 50 genes that contribute to PC1 (D) and PC2 (E).

To provide detailed data on the effect of lipoproteins on global inflammatory responses to *S. pneumoniae*, human MDM genome-wide transcriptional responses were compared after incubation for 4 h with live WT or Δ*lgt* TIGR4 bacteria, or the specific TLR2 agonist Pam_2_CSK_4_. All three stimuli caused major changes in gene expression; Pam_2_CSK_4_ and WT TIGR4 stimulated the increased expression of 854 and 936 genes, respectively, with the Δ*lgt* strain causing upregulated expression of slightly fewer genes (591 in total) (Fig. 6A, 6B). Although there was a large overlap in the genes upregulated by Pam_2_CSK_4_, WT TIGR4, and the Δ*lgt* strain, there was greater overlap with Pam_2_CSK_4_ for responses to WT TIGR4 than for Δ*lgt*, in keeping with the hypothesis that *S. pneumoniae* lipoproteins are important for TLR2-associated transcriptional responses.

PC analysis was used to explore differences in cocorrelated gene expression data. In PC1, which reflects the greatest gene expression differences between stimulated and unstimulated macrophages, the effects of WT TIGR4 were equivalent to those of Pam_2_CSK_4_. However, Δ*lgt*-induced changes in gene expression were reduced for this component (Fig. 6C, 6D). In contrast, the Δ*lgt* and WT TIGR4 strains induced comparable changes to gene expression in PC2, distinct from those induced by Pam_2_CSK_4_ and therefore by inference independent of TLR2 (Fig. 6C, 6E). This analysis is consistent with the Venn diagram of qualitative transcriptional responses to these stimuli, which showed that MDMs stimulated with the Δ*lgt* and TIGR4 strains shared increased expression of 320 genes that were not upregulated following specific TLR2 stimulation by Pam_2_CSK_4_ (Fig. 6B). Bioinformatic analysis of the top 50 genes that reflected *S. pneumoniae* TLR2-dependent responses in PC1 and TLR-2 independent responses in PC2 is presented in Supplemental Fig. 1. Transcription factor binding site enrichment analyses revealed the dominance of NF-κB–regulated genes in PC1, associated with enrichment for proinflammatory cytokines reflected by pathway and gene ontology analysis (Supplemental Fig. 1). In contrast, TLR2-independent responses to *S. pneumoniae* reflected in PC2 showed markedly less enrichment for NF-κB–regulated genes, with modest enrichment for genes associated with nucleosome and transcriptional regulation instead. Taken together, these data suggest that the canonical inflammatory responses to TIGR4 are mediated by TLR2 and are attenuated in response to the Δ*lgt* strain. However, non-TLR2 transcriptional responses were largely comparable in MDMs stimulated with either the Δ*lgt* or TIGR4 strains.

The genes showing the greatest differences in expression between MDMs stimulated with Δ*lgt* and TIGR4 strains included the cytokines IL-23, IL-6, and IL-1β, and the chemokines CXCL1, CXCL2, and CXCL3 (Supplemental Fig. 2A), and transcriptional factor binding site analysis showed enrichment for regulation by NF-κB (Table I) in keeping with the data shown in Fig. 5 and Supplemental Fig. 1. Selected differences in expression of prototypic inflammatory genes identified by microarray were validated by qPCR. *IL-23*, *IL-6*, and *PTGS2* expression were all significantly lower in macrophages stimulated with the Δ*lgt* strain compared with WT TIGR4, confirming the transcriptome results (Supplemental Fig. 2A–E). Although the microarray and qPCR analysis showed no difference in TNF-α expression induced by the two strains (Supplemental Fig. 2A, 2F), both TNF-α and IL-6 production by macrophages were attenuated in response to the Δ*lgt* strain (Supplemental Fig. 2G). Similarly, reduced levels of IL-1β, IL-6, and TNF-α were present in supernatants from human PBMCs stimulated with the Δ*lgt* strain compared with TIGR4 (Table II).

**Table I.** Transcription factor enrichment of gene expression differences between MDMs stimulated with TIGR4 and ∆lgt

Transcription Factor	Background TFBS Rate	Target TFBS Rate	Z-Score*^a^*
RELA	0.0035	0.0103	26.16
NF-κB	0.0050	0.0118	22.11
REL	0.0081	0.0167	21.66
HLF	0.0049	0.0109	19.48
NFKB1	0.0020	0.0053	17.10
NFIL3	0.0033	0.0066	13.19
STAT1	0.0016	0.0038	12.10
Foxq1	0.0060	0.0100	11.71
Ar	0.0006	0.0017	10.32

^*a*^ Z-scores of >10 are considered to indicate highly significant overrepresentation of TFBS within the analyzed gene list.

TFBS, transcription factor binding site.

**Table II.** Mean (SEM) cytokine responses in pg/ml 4 h after incubation of PBMCs from five donors with the TIGR4 wild-type and Δlgt *S. pneumoniae* strains

Cytokine	Conditions				*p* for TIGR4 vs. Δ*lgt*
	Unstimulated	Pam_2_CSK_4_	TIGR4	Δ*lgt*	
IFN-γ	34 (9.6)	94 (16)	56 (16)	38 (9.9)	NS
IL-10	4.7 (1.2)	58 (25)	6.8 (1.7)	16 (10)	NS
IL-12	3.1 (1.3)	9.8 (0.5)	10 (1.1)	6.5 (1.7)	NS
IL-1β	30 (9.9)	1,410 (433)	7,040 (634)	3,260 (501)	0.0016
IL-6	127 (25)	3,340 (797)	245 (57)	98 (18)	0.040
IL-8	5,190 (1,180)	5,210 (715)	10,780 (1,220)	8,650 (1,220)	NS
TNF-α	690 (202)	8,030 (2,160)	4,710 (965)	1,650 (392)	0.019

The *p* values are for comparisons of results obtained with the TIGR4 or *Δlgt* strains (unpaired *t* tests).

### Role of lipoproteins for inflammatory responses to *S. pneumoniae* during infection

**FIGURE 7.**
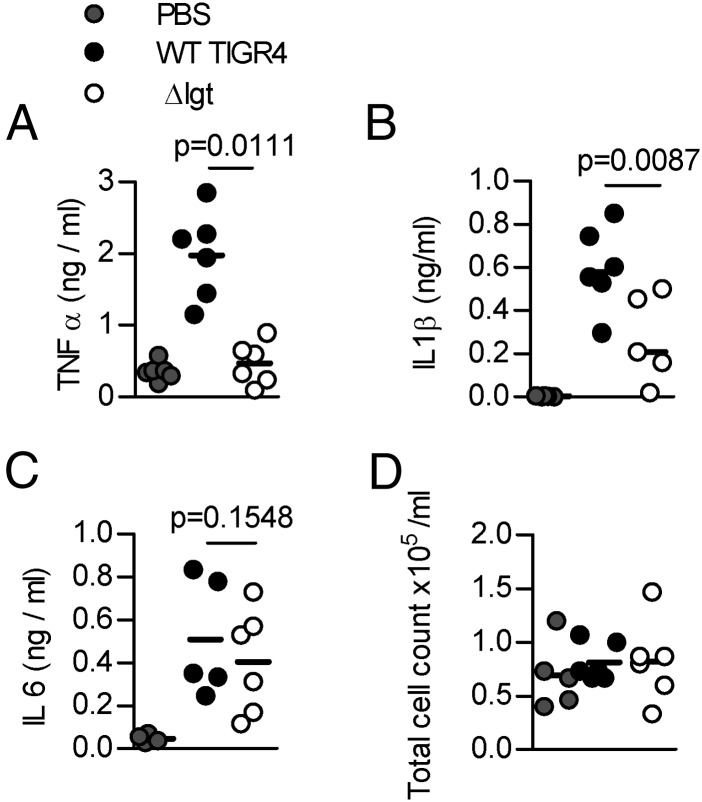
Effects of lipoproteins during early lung infection. CD1 mice (*n* = 5–6) were inoculated intranasally with 1 × 10^7^ CFU TIGR4*ΔpabB* or *Δlgt*/*pabB* and BALF obtained at 4 h. BALF TNF-α (**A**), IL-1β (**B**), and IL-6 (**C**) (PBS control data obtained during a separate infection experiment) levels measured by ELISA. (**D**) BALF total cell count. Each symbol represents results from a single mouse, and the bars medians. The *p* values were obtained using Mann–Whitney *U* tests.

A mouse model was used to investigate the role of lipoproteins for inflammatory responses to *S. pneumoniae* during lung infection. As the *Δlgt* strain cannot replicate efficiently under in vivo conditions and is highly attenuated in virulence (45), only a short-term infection model could be used. To reduce differences in bacterial CFU confounding the results, the experiments were performed in the Δ*pabB* background, which is unable to replicate during infection (47). Four hours after intranasal inoculation with 10^7^ CFU, BALF TNF-α and IL-1β levels were raised in response to the Δ*pabB* strain compared with the Δ*pabB/*Δ*lgt* strain (Fig. 7A, 7B) despite similar BALF CFU (5.14 and 5.16 log_10_ CFU/ml, respectively). BALF IL-6 levels were similar for both strains, although in vitro IL-6 release by BMDMs was dependent on TLR2 and reduced in response to the Δ*lgt* strain (Fig. 7C, Supplemental Fig. 3). Neither strain caused a significant increase in BALF cellularity at this early time point (Fig. 7D).

### IRAK-4–dependent release of protective proinflammatory cytokines from human PBMCs is largely dependent on lipoproteins

**FIGURE 8.**
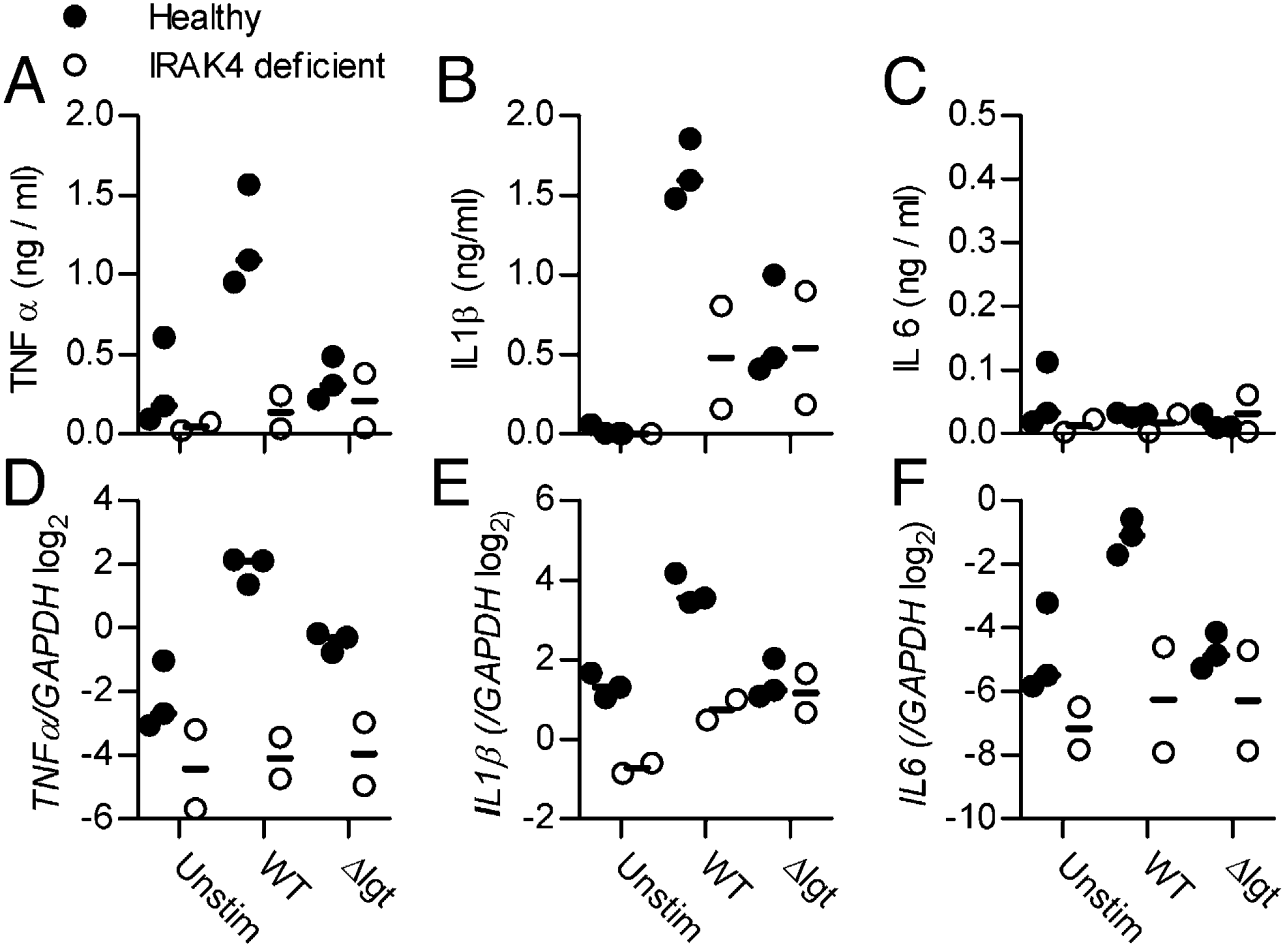
IRAK4-dependent production of proinflammatory cytokines is largely lipoprotein dependent. PBMCs from three healthy volunteers and two IRAK4-deficient patients were stimulated for 4 h with TIGR4 or *Δlgt S. pneumoniae* (MOI 10). (**A**–**C**) Mean TNF-α (A), IL-1β (B), and IL-6 (C) concentrations measured by ELISA in cell culture supernatants. (**D**–**F**) qPCR of *TNF-α* (D), *IL-1β* (E), and *IL-6* (F) gene expression (relative to *GAPDH*). Each symbol represents results from a single donor.

To further assess the clinical relevance of TLR-dependent inflammatory responses to lipoproteins, the WT TIGR4 and Δ*lgt* strains were incubated with PBMCs obtained from two humans with IRAK-4 deficiency, and cytokine transcriptional and protein responses were measured. High levels of TNF-α and IL-1β were found in supernatants from healthy control PBMCs incubated with WT TIGR4. In contrast, incubation of healthy control PBMCs with the Δ*lgt* strain or IRAK-4^−/−^ PBMCs with either WT or Δ*lgt* TIGR4 resulted in similar reduced levels of TNF-α and IL-1β (Fig. 8A, 8B). In these experiments, no significant PBMC IL-6 response was detected (Fig. 8C). qPCR demonstrated increased expression of *TNFα*, *IL1-β*, and *IL-6* by control PBMCs in response to WT TIGR4, a response that was significantly attenuated in PBMCs from IRAK-4–deficient individuals (Fig. 8D–F). In contrast, there was no induction of *IL-1β* and *IL-6* gene expression by the Δ*lgt* strain in either control or IRAK-4–deficient PBMCs (Fig. 8E, 8F) and reduced *TNF-α* expression by control PBMCs (Fig. 8D). Overall, these data show that important components of the IRAK-4–dependent inflammatory response to *S. pneumoniae* are considerably reduced in response to the Δ*lgt* strain, indicating that lipoproteins are likely to be major stimuli for IRAK-4–dependent immune responses.

## Discussion

*S. pneumoniae* infections cause a strong NF-κB–mediated proinflammatory response that is essential for host immunity but also causes some of the important features of severe infection (2–4, 60). This inflammatory response has previously been shown to be partially dependent on TLR2 (18, 19, 23, 24, 61). In addition, TLR2 responses are required for Th17 and some humoral adaptive immune responses (19, 28, 29). Identifying the *S. pneumoniae* TLR2 ligands is important for our understanding of disease pathogenesis and for the design of therapeutic manipulations aimed at modifying inflammatory responses or improving humoral and cellular immunity to *S. pneumoniae*. Although the cell wall components PGN and LTA are proinflammatory *S. pneumoniae* ligands thought to be recognized by TLR2 (33–35), recent evidence suggests they may stimulate inflammatory responses by TLR2-independent mechanisms (36–38). In contrast to the extensive data on PGN and LTA, the potential role of lipoproteins as proinflammatory stimuli during *S. pneumoniae* infections has not been characterized. We have now explored the role of lipoproteins as *S. pneumoniae* proinflammatory ligands using the Δ*lgt* strain (45), which allows the effects of lipoproteins to be assessed in the context of the whole bacterium rather than relying on purified products.

Our results demonstrated that *S. pneumoniae* lipoproteins are major contributors to the macrophage TLR- and NF-κB–mediated inflammatory response. In theory, the effects of the Δ*lgt* mutation on the physiology of *S. pneumoniae* (45) could have confounded these results, but control experiments demonstrated persistent decreases in macrophage inflammatory responses to the Δ*lgt* strain when live or nonreplicating (to control for bacterial CFU) bacteria were used. The data obtained by the nonreplicating *ΔpabB* strains could also have been confounded by the dual mutation in the *Δlgt/pabB* strain compared with the *ΔpabB* strain; however, additional control experiments also showed reduced inflammatory responses to lipoprotein extracts or sonicated bacteria (to release cell wall fragments) from the *Δlgt* strain compared with WT. Differences in TNF-α responses to TIGR4 and Δ*lgt* strains were lost for macrophages from TLR2 mice and preserved for macrophages from TLR4 mice. Of the surface structures that affect interactions with the host, *S. pneumoniae* LTA has recently been shown not to affect TLR2 responses (38), and we have shown no effect of the Δ*lgt* mutation on PGN structure and (in a previous publication) on neutrophil killing of *S. pneumoniae*, a phenotype suggesting the capsule is unaffected (45). Furthermore, two papers have shown the capsule has limited effects on inflammation (62, 63), and, although a third suggests capsule material can induce inflammatory responses (64), these were measured at a much later time point than our data and may be confounded by contamination of capsule polysaccharide with cell wall or lipoprotein. Hence, it is unlikely that the differences in inflammatory responses seen between WT and Δ*lgt* strains were confounded by effects of the mutation on bacterial physiology, PGN structure, or the capsule. Complementing the *Δlgt* mutation would have been beneficial, but for poorly understood reasons *S. pneumoniae* mutant strains can be difficult to complement, and our attempts to complement the *Δlgt* mutant failed (45). Instead, we used whole-genome sequencing and transfer of the mutation to another strain to link the phenotypes seen to deletion of *lgt*. These results indicate that lipoproteins are major TLR2 ligands for *S. pneumoniae*, similar to results obtained with other Gram-positive pathogens (39, 40, 51, 65).

We used transcriptional arrays, qPCR, and supernatant cytokine levels to confirm decreased expression of a variety of proinflammatory cytokines and chemokines for macrophages infected with the Δ*lgt* strain. There were some discrepancies between transcriptional and protein level data with, for example, consistently reduced levels of TNF-α protein in response to infection of MDMs, BMDMs, and PBMCs despite little change in gene transcription at the 4-h time point, which may reflect release of preformed TNF-α. In addition, significant levels of IL-1β, IL-6, and TNF-α were still produced by leukocytes in response to the *Δlgt* strain, potentially reflecting inflammatory responses induced by NOD2-mediated recognition of PGN and pneumolysin activation of the inflammasome and/or TLR4 (16, 27, 36, 37, 66, 67). These mechanisms combined with residual TLR2 activation may explain why the macrophage proinflammatory gene transcriptional responses to the Δ*lgt* strain were reduced rather than absent compared with WT TIGR4. These results are also consistent with published data showing more marked effects of MyD88 deficiency than loss of individual TLRs on inflammatory response to *S. pneumoniae* in mice and humans (8, 11, 17, 19, 21). As TLR2 activation increases TLR4-, pneumolysin-, and NOD2-dependent inflammatory responses to *S. pneumoniae* (21, 26, 27), *S. pneumoniae* lipoproteins may also increase inflammatory responses indirectly through these other mechanisms of innate immune recognition.

The global assessment of macrophage responses by transcriptional profiling significantly improves our understanding of key interactions of the host with *S. pneumoniae*. *S. pneumoniae* caused major changes in macrophage gene expression, with upregulation of >900 genes in response to the WT TIGR4 strain. Many genes showing large differences in expression between control macrophages and those infected with TIGR4 *S. pneumoniae* were equally expressed in response to infection with the *Δlgt* strain. However, the small proportion of genes with differences in expression between the WT and *Δlgt* strain was concentrated in the transcriptional responses with the greatest upregulation in response to *S. pneumoniae* (PC1), which also responded to Pam_2_CSK_4_ stimulation. These data show that macrophage transcriptional responses to *S. pneumoniae* are dominated by TLR2-dependent genes that often require the presence of lipoproteins for maximal stimulation, with a larger number of generally weaker changes in expression of genes whose responses are TLR2 independent. The list of TLR2-dependent genes has a striking preponderance for genes encoding important proinflammatory proteins that bioinformatic analysis suggests are largely activated by the NF-κB pathway, in keeping with the data showing reduced NF-κB activation in response to the *Δlgt* strain. Our data contrast with those showing that pneumolysin induced macrophage expression of very few genes encoding chemokines or cytokines (68).

The potential clinical relevance of *S. pneumoniae* lipoprotein-dependent inflammation was assessed in a mouse model of pneumonia, and using PBMCs from individuals with IRAK-4 deficiency. We were able to demonstrate that the rapid TNF-α and IL-1β responses during early lung infection with *S. pneumoniae* were reduced in response to the Δ*lgt* strain. Despite the effect of lipoproteins on the IL-6 response in vitro, BALF IL-6 responses were similar for mice infected with the TIGR4 or Δ*lgt* strain. Potentially, differences in IL-6 levels could develop later in infection, but as the Δl*gt* strain cannot maintain long-term infection in mouse models (45), it was not feasible to investigate this. The striking susceptibility of children with IRAK-4 deficiency to invasive *S. pneumoniae* infections demonstrates the vital importance of inflammatory responses for immunity to this pathogen during childhood (5, 6, 9, 10), but the *S. pneumoniae* ligands responsible have not been identified. In mouse models of infection, IL-1β, IL-6, and TNF-α are important protective cytokines during *S. pneumoniae* infection (13–15), and we have now demonstrated that expression of these cytokines by healthy controls was largely dependent on lipoproteins. Importantly, WT PBMCs IL-1β, IL-6, and TNF-α responses to the Δ*lgt* strain were attenuated at the transcriptional and (for IL-1β and TNF-α) protein level, similar to the results for WT TIGR4 incubated with PBMCs obtained from IRAK4-deficient subjects. Due to the limited number of donors available, only two IRAK4-deficient subjects were available for testing. Despite this, the results were highly consistent between the two donors and suggest that lipoproteins are important ligands driving IRAK-4–dependent inflammatory responses and therefore protective immunity in children.

Overall, our data demonstrate that lipoproteins are major *S. pneumoniae* TLR2 ligands that are required for the maximum transcriptional response for many of the dominant macrophage gene responses to *S. pneumoniae*, including induction of IRAK-4–dependent protective cytokines. Specific targeting of bacterial lipoprotein/TLR2 inflammatory responses could be a novel therapeutic approach for enhancing cellular and humoral immune responses to vaccines or, when combined with effective antibiotic therapy, for improving the outcome of severe *S. pneumoniae* infections.

## Supplementary Material

Data Supplement
