# Supplementary material for: TLR-Mediated Inflammatory Responses to Streptococcus pneumoniae Are Highly Dependent on Surface Expression of Bacterial Lipoproteins
Source: J Immunol. 2014 Aug 29;193(7):3736–45. doi: 10.4049/jimmunol.1401413 (PMC4170674; doi:10.4049/jimmunol.1401413)
Supplement: Data Supplement [file supp_193_7_3736__index.html]

TLR-Mediated Inflammatory Responses to Streptococcus pneumoniae Are Highly Dependent on Surface Expression of Bacterial Lipoproteins — Data Supplement 

# TLR-Mediated Inflammatory Responses to *Streptococcus pneumoniae* Are Highly Dependent on Surface Expression of Bacterial Lipoproteins

## Data Supplement

**Files in this Data Supplement:**

- Supplemental Material 1 (PDF)
