# Supplementary material for: TLR-Mediated Inflammatory Responses to Streptococcus pneumoniae Are Highly Dependent on Surface Expression of Bacterial Lipoproteins
Source: J Immunol. 2014 Aug 29;193(7):3736–45. doi: 10.4049/jimmunol.1401413 (PMC4170674; doi:10.4049/jimmunol.1401413)
Supplement: Data Supplement [file 1401413_JI_1401413_Supplemental_Material_1.pdf]

Supplementary Table 1.

Single nucleotide polymorphisms identified in the genome sequences of the TIGR4 and D39 *Δlgt* strains compared to their parental strains.

**Genome position**	**Gene affected**	**Reference bp / codon**	**Mutant bp / codon**	**Notes**
**D39*Δlgt***				
1272512	SPD_1244 *hprK*	ATC	ATA	Synonymous mutation
1272548	SPD_1244 *hprK*	GCG	GCT	Synonymous mutation
**TIGR4*Δlgt***				
730678	Intergenic region	C	T	n/a
1332449	SP_1413 *hprK*	GCG	GCT	Synonymous mutation
1332584	SP_1413 *hprK*	AAT	AAC	Synonymous mutation
2092182	SP_2175 *dltB*	CAT	CGT	Non-synonymous mutation: changes a histidine for an arginine at position 336 of 413

Supplementary Fig. 1

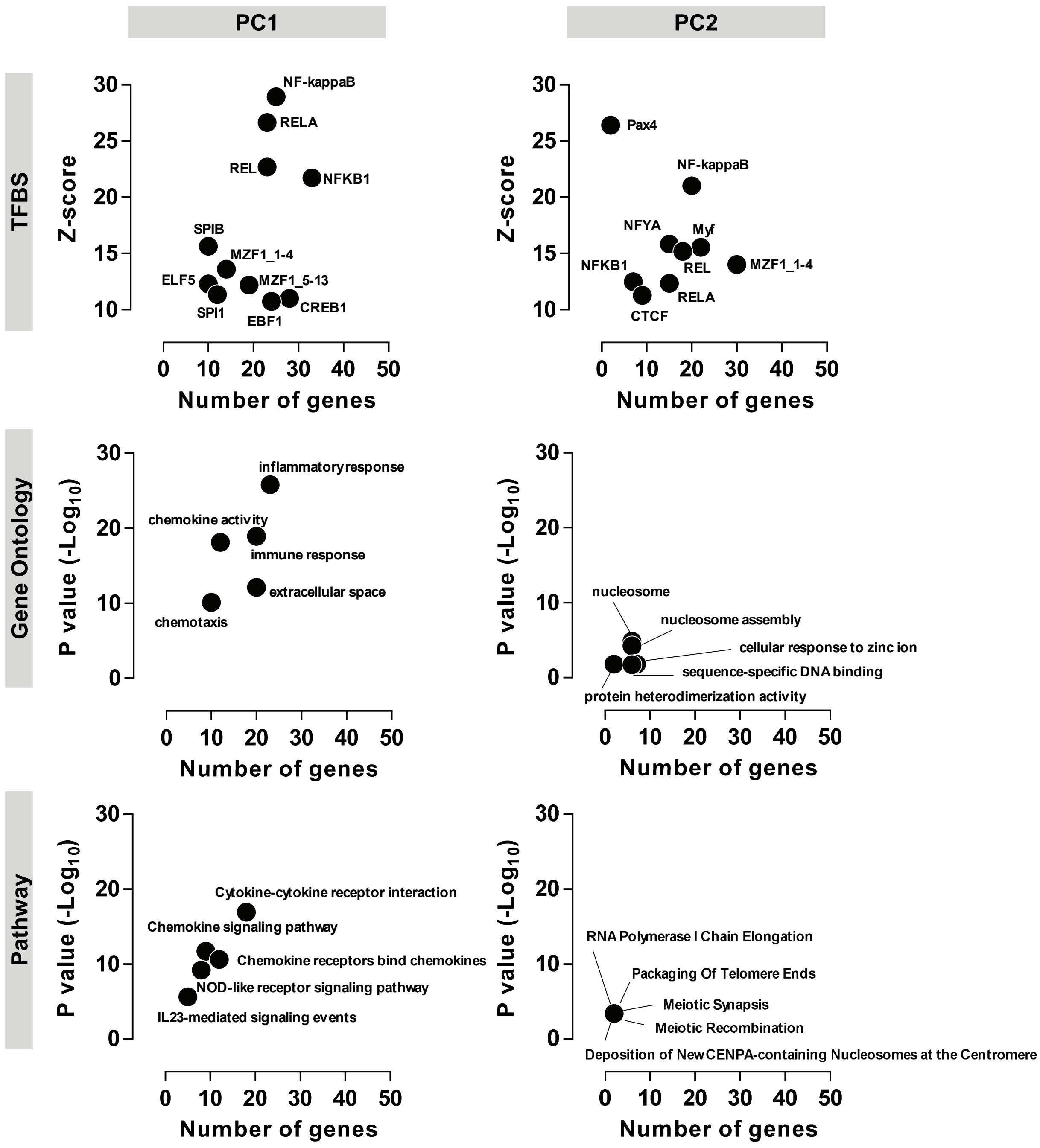

The top 50 transcriptional responses associated with principal component (PC)1 and PC2 in Figure 6D-E were subjected to transcription factor binding site (TFBS) enrichment analysis using oPossum (http://opossum.cisreg.ca/oPOSSUM3/), and to Gene Ontology and Pathway enrichment analyses using innateDB (http://www.innatedb.com/). In each case the test statistic (Z-score or corrected P value) and the number of genes associated with each annotation is presented as an individual data point. A Z-score >10 and $-\text{Log}_{10}$ P>1.3 are considered statistically significant.

Supplementary Fig. 2

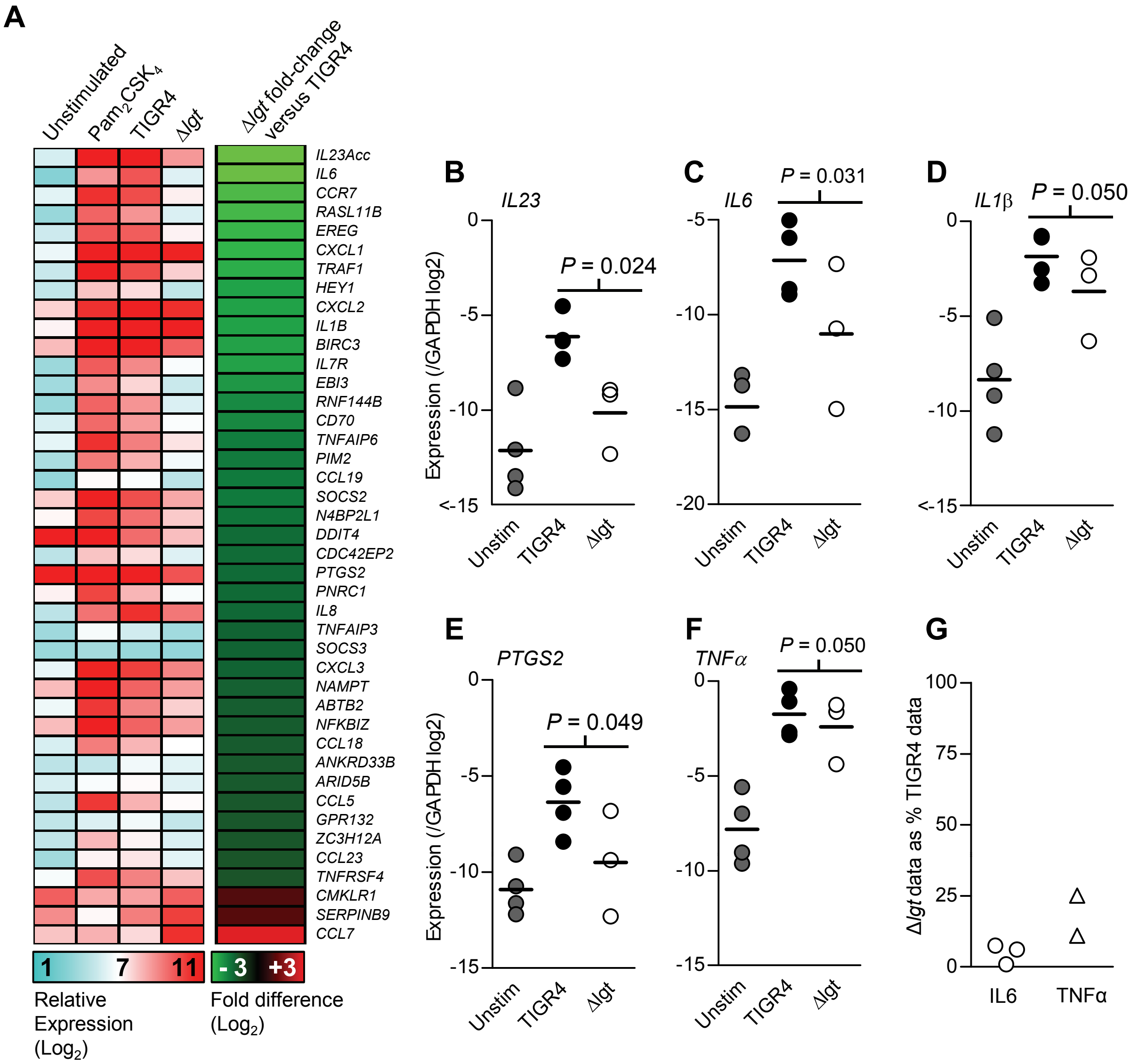

(A) Gene expression heat map for mean relative gene expression levels and fold differences for the 42 genes showing significant differences in expression ($P < 0.05$) between human monocyte derived macrophages (MDM) from at least three different donors stimulated for four hours with TIGR4 or *Δlgt S. pneumoniae*. (B-F) Validation using qPCR (normalised to GADPH expression) of selected pro-inflammatory genes showing reduced expression in the transcriptional array analysis after incubation with *Δlgt* strain compared to TIGR4. (G) Relative supernatant levels of IL-6 and TNFα (measured using a luminex system) in cell culture supernatants of MDMs incubated with the *Δlgt* strain expressed as a percentage of the result for the same donor when incubated with the TIGR4 strain. For panels (B) to (G) each symbol represents results from a single donor, and the bars medians. *P* values are for comparisons of results for MDM stimulated with the TIGR4 or *Δlgt* strains (paired t tests).

Supplementary Fig. 3

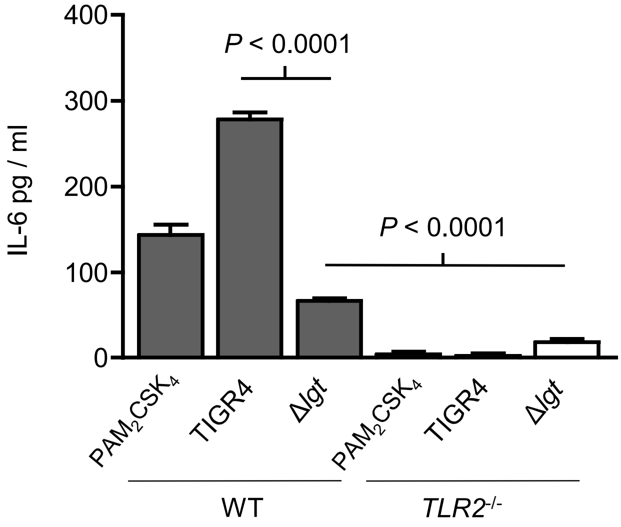

IL6 concentrations in cell culture supernatants from BMDMs obtained from C57BL/6 and *TLR2*$^{-/-}$ mice incubated for 4 hours with the TLR2 agonist Pam_2CSK_4, or TIGR4 or TIGR4*Δlgt S. pneumoniae* strains. n = 3 to 4, error bars represent SEMs, and the stated *P* values were obtained using unpaired t tests.
